# Comparative Study of Dermal Pharmacokinetics Between Topical Drugs Using Open Flow Microperfusion in a Pig Model

**DOI:** 10.1007/s11095-023-03645-3

**Published:** 2023-12-29

**Authors:** Manfred Bodenlenz, Thean Yeoh, Gabriel Berstein, Shibin Mathew, Jaymin Shah, Christopher Banfield, Brett Hollingshead, Stefanus J. Steyn, Sarah M. Osgood, Kevin Beaumont, Sonja Kainz, Christian Holeček, Gert Trausinger, Reingard Raml, Thomas Birngruber

**Affiliations:** 1 HEALTH - Institute for Biomedical Research and Technologies, Joanneum Research Forschungsgesellschaft M.B.H, Neue Stiftingtalstrasse 2, 8010 Graz, Austria; 2 Pfizer Research Technology Center, 1 Portland St, Cambridge, MA 02139 USA

**Keywords:** candidates, clinical efficacy, compounds, free drug hypothesis, *in vivo* animal model

## Abstract

**Purpose:**

Accurate methods to determine dermal pharmacokinetics are important to increase the rate of clinical success in topical drug development. We investigated in an *in vivo* pig model whether the unbound drug concentration in the interstitial fluid as determined by dermal open flow microperfusion (dOFM) is a more reliable measure of dermal exposure compared to dermal biopsies for seven prescription or investigational drugs. In addition, we verified standard dOFM measurement using a recirculation approach and compared dosing frequencies (QD *versus* BID) and dose strengths (high *versus* low drug concentrations).

**Methods:**

Domestic pigs were topically administered seven different drugs twice daily in two studies. On day 7, drug exposures in the dermis were assessed in two ways: (1) dOFM provided the total and unbound drug concentrations in dermal interstitial fluid, and (2) clean punch biopsies after heat separation provided the total concentrations in the upper and lower dermis.

**Results:**

dOFM showed sufficient intra-study precision to distinguish interstitial fluid concentrations between different drugs, dose frequencies and dose strengths, and had good reproducibility between studies. Biopsy concentrations showed much higher and more variable values. Standard dOFM measurements were consistent with values obtained with the recirculation approach.

**Conclusions:**

dOFM pig model is a robust and reproducible method to directly determine topical drug concentration in dermal interstitial fluid. Dermal biopsies were a less reliable measure of dermal exposure due to possible contributions from drug bound to tissue and drug associated with skin appendages.

**Supplementary Information:**

The online version contains supplementary material available at 10.1007/s11095-023-03645-3.

## Introduction

The cost of clinical failure represents a significant proportion of the overall R&D development costs [1, 2]. As such, R&D productivity is customarily measured by the success rate. The success rate for the mid and late stage (phase 2 and 3) clinical studies is the most crucial as these stages are more costly and have the longest duration. Therefore, rapidly progressing programs with strong supportive enabling data while stopping weaker programs earlier would be expected to increase overall R&D productivity [3].

In addition to ensuring that a drug has the desired target modulation properties during the development of topical New Chemical Entity (NCEs) drugs, it is also essential to ensure that sufficient drug concentrations can be achieved in the targeted skin tissue compartments. Such knowledge of target skin tissue concentrations is key for a more rational drug development process [4, 5]. Preclinical models offer the possibility to assess whether the formulated drug product can deliver and achieve sufficient drug concentrations in the target tissue. Pig models have been extensively studied for the use in *in vivo* percutaneous absorption/penetration of formulated drugs as pig skin is considered most similar to human skin in terms of skin anatomy and permeability characteristics [6–9]. Ideally, such preclinical models should be able to reflect human skin pharmacokinetics (PK) and pharmacodynamics (PD) under *in vivo* conditions where active drug absorption and clearance are occurring. Any sampling method used in *in vivo* conditions should ideally be able to measure the pharmacologically active unbound drug concentrations in the local tissue, preferably at the drug’s target site, according to the free drug hypothesis [10–13]. Dermal microdialysis (dMD) has been used to measure the rate and extent of absorption of topically administered drugs in humans and animals [6, 14], including pigs and minipigs [6, 15]. However, dMD has several drawbacks, including a major limitation in accurate sampling of lipophilic drugs presumably due to high protein binding [14, 16–18].

Open flow microperfusion (OFM) has been developed as an alternative sampling technology to enable direct measurement of drug and biomarker concentrations in target tissues by sampling interstitial fluid (ISF) under *in vivo* conditions in humans and animals. As a minimally-invasive and well-tolerable method, it has been successfully utilized in human clinical studies to investigate biomarkers and drug PK-PD in target tissues in healthy subjects and patients [19–24]. In preclinical research it has been utilized for the study of novel compounds at their targets in animal brain [25–27] and in animal peripheral tissues *in vivo* [28–31] as well as in explanted human and animal tissues *ex vivo* [32, 33]. The probes for dermal OFM (dOFM probes) provide access to dermal interstitial fluid (dISF) for the continuous *in vivo* assessment of drugs and biomarker concentrations in humans and animals [17, 28, 34–38]. dOFM probes have been utilized in preclinical research to clarify differences between topical drugs. In one study, freshly excised human skin was used to show that dOFM but not biopsy measurements explained the difference in clinical activity between two PDE4 inhibitors [32]. In another preclinical pig study, dOFM and biopsies combined with MALDI-MSI were used to explain differences in the efficacies of two JAK inhibitors [33].

The present study aimed to compare dermal drug concentrations by using dOFM and biopsies in pigs *in vivo* after a one-week application of several topical prescription and investigational drugs. This study also included basic methodological assessments such as intra-study precision of both methods, between-study reproducibility of dOFM, and a verification of the dOFM concentrations using an orthogonal approach.

## Materials and Methods

### Drugs and Formulations

Seven different compounds were selected for topical dose administration in two pig studies (Table I) including prescription drugs and investigational drugs. These seven drugs were selected because they cover a wide range of drug properties (MW, clogP, protein binding) and because data are available on their clinical and pharmacological activity (Table II, plus Supplementary Material provides summary of clinical efficacy data) which facilitates comparative assessment of pig PK data to the drugs’ clinical activity. Four additional compounds were studied as part of pig Study#2 but were not disclosed herein as no clinical data are available for the intended assessment.

**Table I:** Test Article Description and Dosing, BID (twice a day), QD (once a day)

Test Article Description	Study #	Application Rate	Application Frequency
Brepocitinib 3% cream	1, 2	2.0 mg/cm^2^/dose	BID, QD in Study#2
Brepocitinib 0.3% cream	1	2.0 mg/cm^2^/dose	BID
Tofacitinib 2% ointment	1	3.0 mg/cm^2^/dose	BID
Crisaborole 2% ointment	1	3.0 mg/cm^2^/dose	BID
PF-06763809 2.3% solution	1, 2	2.5 µL/cm^2^/dose	BID
PF-06263276 4% solution	1	2.5 µL/cm^2^/dose	BID
Diclofenac Diethylamine 2% gel	1	2.5 mg/cm^2^/dose	BID
Ruxolitinib 1.5% cream	2	2.0 mg/cm^2^/dose	BID

Brepocitinib, tofacitinib, crisaborole, PF-06763809 and PF-06263276 were manufactured by Pfizer. Brepocitinib 3% and 0.3% creams, tofacitinib 2% ointment and crisaborole 2% ointment formulations were prepared by Pfizer (USA). PF-06763809 2.3% solution and PF-06263276 4% solution were prepared by Joanneum Research according to formulation instructions provided by Pfizer. The formulations for the drugs were developed with consideration for optimal skin penetration and acceptable product stability. The brepocitinib cream formulation vehicle was the same for the 3% and 0.3% strengths in order to test the influence of the concentration. Diclofenac diethylamine (Voltadol Forte 2% gel) was included as a reference test article (sourced in Austria). Ruxolitinib 1.5% cream was prepared by Pfizer according to a published protocol [39]. Ruxolitinib phosphate was purchased from Advanced ChemBlocks (Hayward, CA, USA). The inactive ingredients of the formulations prepared for this study were of compendial grade.

### *In vivo* PK Studies

#### Animals

Six young domestic male castrated Landrace pigs (5–7 weeks, Source: H. Stelzl, Austria) were investigated in each of the two studies. Pigs were delivered to the Division of Biomedical Research (Medical University of Graz, Austria) and trained by the staff for 1 to 2 weeks prior to the study to accustom them to a restraining hammock for topical dose administration. The body weight on day 1 was 12–17 kg for Study#1 and 9–14 kg for Study#2.

#### Topical Treatments on Days 1–7

Before the start of topical application, hair at the application sites was carefully clipped.

**Fig. 1:**
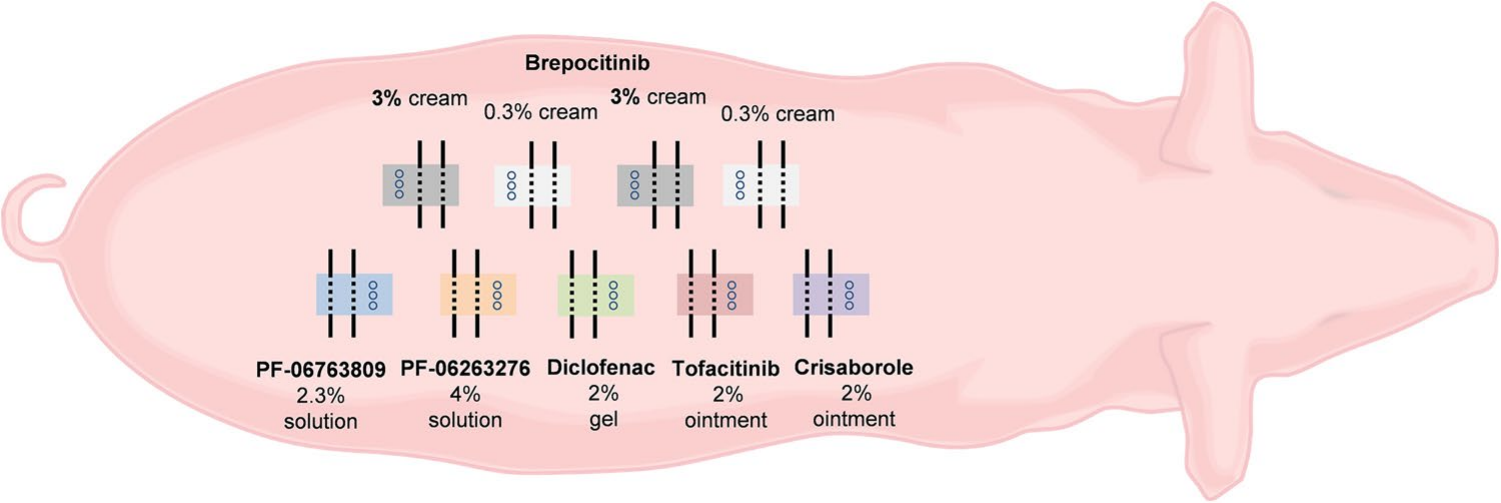
Study setting for pigs #1 to #6 in Study#1 (randomization not shown). The application sites (2.5 × 4.5 cm) were arranged in two rows 3 cm to the left and to the right of the spine. The setting of Study#2 was comparable but did not include skin biopsies and thus the size of each application site was reduced to 2.5 × 3.2 cm. Circles indicate biopsy sites and lines indicate dOFM probes (two probes/application site).

On day 1 of the study, pigs were anesthetized by using an isoflurane inhalation protocol. The skin was cleaned with gauze that was soaked in water. Nine application sites (2.5 × 4.5 cm in Study#1; 2.5 × 3.2 cm in Study#2) were demarcated with a permanent marker on the back of each animal (Fig. 1). dOFM probe puncture sites (planned for probe insertions on day 7) were sealed with Kryolan silicone adhesive and covered with a transparent medical film (Opsite flexifix, Smith + Nephew, UK) to avoid contamination with the test formulations. Formulations were then topically applied (Table I). The six semi-solid formulations were applied with pre-weighed spatulas and distributed evenly over the application site with a finger cot. The two liquid formulations were applied with positive displacement pipettes and distributed with the pipette tip and a finger cot. All drugs were applied twice daily (BID, in the early morning and late afternoon) with approximately 12 h between applications except for brepocitinib in Study#2 which was applied twice daily and once daily (QD, in the early morning) on different applications sites. After dosing, the application sites were covered using a combination of a non-occlusive soft silicone dressing (Mepilex®Lite, Mölnlyke Health Care AB, Sweden) and a non-occlusive transparent film dressing (Tegaderm™, 3 M Health Care, Germany) to avoid any damage or contamination of the skin. Subsequent dose applications on days 1 to 6 were conducted on conscious hammock-restrained animals. Drug applications were randomized among the nine test sites. The last dose was applied on day 7 to anesthetized animals at the beginning of PK investigation.

#### PK Investigation on Day 7

**Fig. 2:**
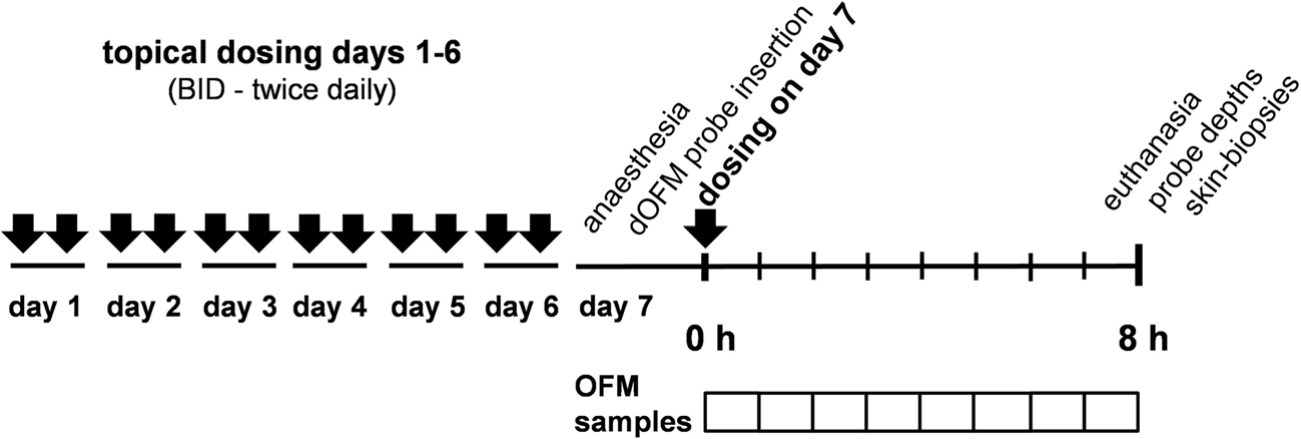
Application and PK sampling schedule. Dosing time of dose #13 (dosing at day 7) is defined as t = 0 h

PK was investigated on day 7 in anesthetized pigs 0–8 h after the last drug application (Fig. 2).

Anesthesia was started with a premedication mixture of midazolam (0.5 mg/kg), azaperone (2.5 mg/kg), ketamine (10 mg/kg), and butorphanol (0.2 mg/kg). The induction of anesthesia was conducted after sufficient preoxygenation with propofol 1% (3 mg/kg bolus). Anesthesia was maintained using propofol 1% (2–5 mg/kg/h), fentanyl (20 µg/kg/h), and—if necessary—isoflurane gas 2%. Furthermore, an isotonic electrolyte solution was administered at a rate of approximately 10 mL/kg/h during the first 60 min of anesthesia followed by 3 mL/kg/h.

For dOFM sampling the protective cover was removed from the topical applications sites. To avoid contamination of the dOFM probes with residual drug on the skin surface while inserting the probes, the needle puncture sites next to the application sites were cleaned following a defined procedure: first, the skin next to the sites was cleaned using gauze soaked with water; second, after drying the skin with gauze, tape stripping was performed 10 times with highly adhesive tape to remove the potentially contaminated layers of stratum corneum at the probe puncture sites.

### dOFM Sampling

Preclinical dOFM probes (a/d OFM-P-15, JR-HEALTH, Graz, Austria; a linear PEEK-type probe with a 15 mm open exchange section) were inserted in the dermis through standard hollow needles (Sterican 20G, B. Braun, Germany) consistent with the insertion practice used clinically. dOFM probes were perfused using small wearable clinical dOFM pumps (MPP102/MPP102 PK, JR-HEALTH; multichannel peristaltic push–pull type) in combination with required accessories (Tubing-kits OFM-PS3-75 and OFM-PL3-75, and OFM-BAG 10 mL, JR-HEALTH, Austria). The perfusate consisted of standard clinical dOFM perfusion fluid (ELO-MEL isotone, Fresenius Kabi, Austria) with 2% human serum albumin (HSA solution 20%, CSL Behring GmbH, Germany). dISF samples were collected directly at the probe outlet into small low-bind sampling vials (0.2 mL PCR tubes, MAXYMUM Recovery, Axygen, USA). Prior to the pig studies, dOFM material and perfusion fluid were qualified in an *in vitro* test by verifying that the study drugs did not encounter significant non-specific adsorption loss as they passed through the probes and were collected in sampling vials.

After probe insertion, the puncture sites were sealed by cyanoacrylate (Cyanolit 241F, Panacol, Germany) thus also fixing the probes in place. dOFM sampling was initiated by flushing the dOFM probes for 5 min at a flow rate of 10 µL/min, then reducing the flow to the nominal rate of 1 µL/min, thus delivering dISF samples of ~ 60 μL/h. The sample vials were exchanged hourly for 8 h and individual samples were weighed as a quality control of the flow rate. Samples from two dOFM probes per application site were pooled hourly for 8 h, thus obtaining eight pooled samples for analysis per site (~ 120 μL/pooled sample). Pooled samples were stored at -20°C until the end of dOFM sampling and then transferred to -80°C until bioanalysis. At termination of dOFM sampling at 8 h, the correct intradermal insertion and depth of each probe was verified by using 22 MHz ultrasound (LOGIQ e R6, GE Healthcare, UK).

### Skin Biopsies

After 8 h of dOFM sampling and euthanasia on day 7, the epidermis was completely removed by heat separation and three 6 mm punch biopsies were taken from the dermis at each application site. To minimize contamination, new disposable biopsy punch, scalpel blade, and forceps were used for each single dermis biopsy. Prior to bioanalysis, the frozen dermal samples were split into upper and lower dermis. A detailed description of the biopsy procedure is provided in the Supplementary Material.

The biopsy results were described by the geometric mean values (GM) of the concentrations for upper dermis and the lower dermis (N = 36 for brepocitinib, N = 18 for other compounds). 

### dISF Drug Concentrations 

The total and unbound dISF drug concentrations were calculated by using an equation that accounted for the dilution factor, the recovery based on diffusivity, and the experimentally determined protein binding. The calculation considers that dISF concentrations were diluted by dOFM probe perfusion with a perfusate, with the degree of dilution described by the “Relative Recovery—RR” [40, 41]. In brief, the RR for a drug primarily depends on the drug's diffusivity in dISF, the probe’s total exchange area, and the perfusion flow rate. The calculation considers that the dOFM probe lacks a filtering semi-permeable membrane and thus recovers both, (i) the small and highly-diffusive unbound drug with a high RR and (ii) the large and much less diffusive protein-bound drug with a low RR. We used well-established empirical values for the RRs of the drug fractions (RR_u_ = 40% for the unbound fraction f_u_, RR_b_ = 10% for the protein-bound fraction f_b_), values which had been obtained by using the same probe type and flow rate in the dermis from several prior *in vivo* studies (unpublished). The values for the two drug fractions (f_b_ + f_u_ = 1) were obtained by small volume RED (Rapid Equilibrium Dialysis) [42] in dISF-like protein-solutions as described above and delivered binding data similar or lower compared to the published plasma binding values.

The total relative recovery RR_tot_ for each individual drug (unbound + bound) into the dOFM sample is defined as:

1 $${{\text{RR}}}_{{\text{tot}}} = {{\text{RR}}}_{{\text{u}}} * {{\text{f}}}_{{\text{u}}} + {{\text{RR}}}_{{\text{b}}} *{{\text{f}}}_{{\text{b}}}$$

The original undiluted total dermal interstitial concentration dISF_tot_ and thereof the undiluted unbound fraction dISF_u_ was then calculated as:

2 $${{\text{dISF}}}_{{\text{tot}}} = {{\text{dOFM}}}_{{\text{tot}}} / {{\text{RR}}}_{{\text{tot}}}$$

3 $${{\text{dISF}}}_{{\text{u}}} = {{\text{dISF}}}_{{\text{tot}}} * {{\text{f}}}_{{\text{u}}}$$

**Fig. 3:**
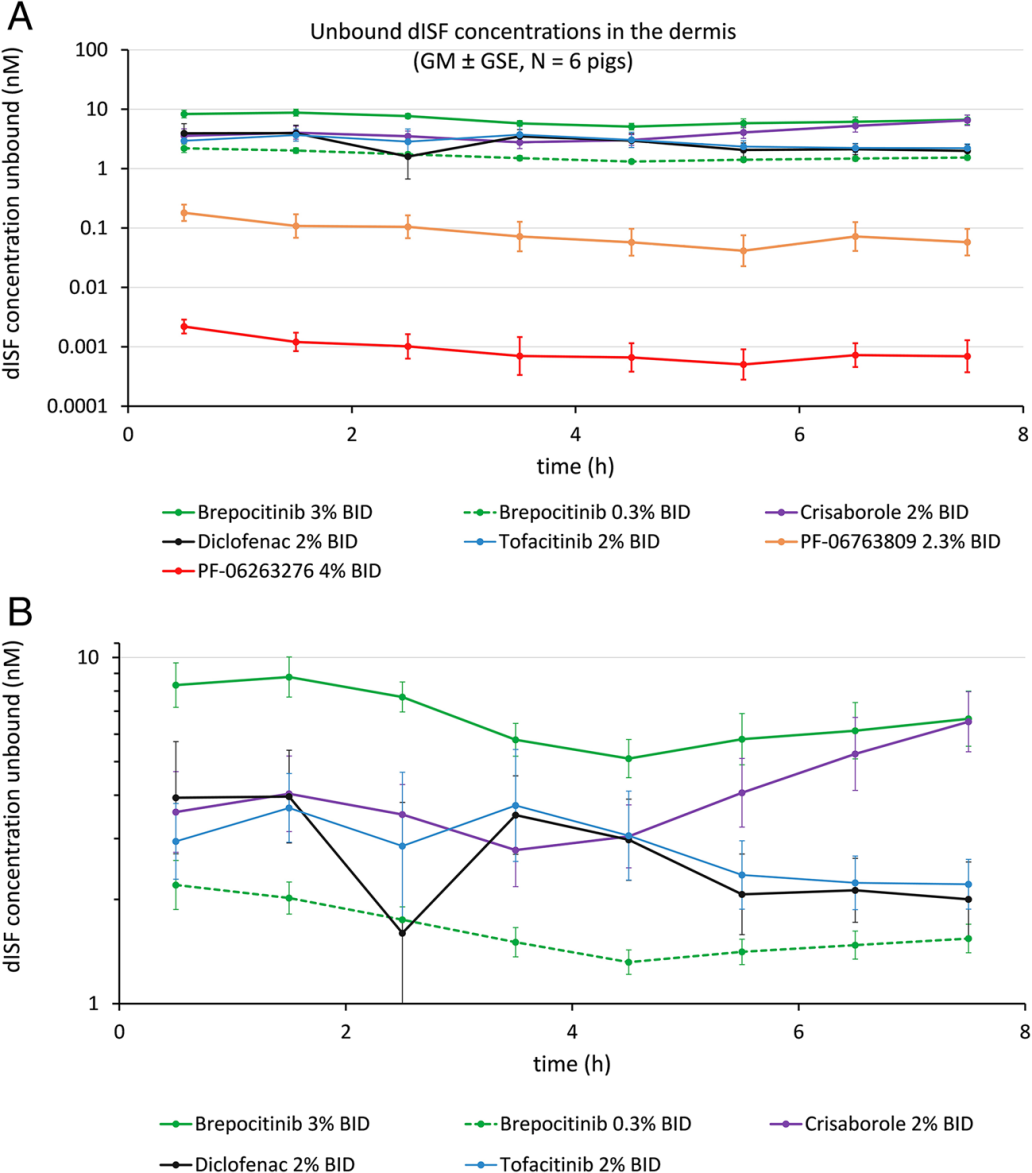
(**A**) Dermal PK profiles of seven tested drugs in Study #1. Drugs included brepocitinib in two formulation strengths, tofacitinib, crisaborole, diclofenac, PF-06763809 and PF-06263276. (**B)** Enlarged PK profiles of five drugs in Study #1. Drugs included brepocitinib in two formulation strengths, tofacitinib, crisaborole, diclofenac

The dISF unbound concentrations were plotted *versus* time as GM ± GSE concentration profiles (geometric mean ± geometric standard error). The GM and GSE were calculated in MS Excel from log-transformed data. The non-symmetric error bars for the GSE were calculated as described on the website of Graphpad (https://www.graphpad.com/support/faq/plotting-the-geometric-mean-with-geometric-sd-error-bars/). The data are plotted on the log-scale to be able to show the profiles for all drugs in one plot (Fig. 3 to Fig. 8).

The dOFM and dISF results were described by the GM of the average concentrations over 0–8 h, and the results for all compounds listed in a table format with declining dISF total concentrations to compare with biopsy results and clinical evidence. The results were obtained for qualitative comparison; concentration differences between compounds/treatments were not evaluated for statistical significance.

### Precision of the Methods

The precision of dOFM and biopsies was evaluated separately in each pig by calculating the coefficient of variation (CV) of the log-transformed PK results obtained for the 8 h time point. For correct calculation of the CV for log-transformed data the equation of Nelson *et al*. was used [43, 44]. The average CV% (mean and median, N = 6 pigs) was used as measure for the precision. This evaluation was based on the brepocitinib data obtained for the 3% and the 0.3% cream BID applications, because these two applications were investigated with more dOFM probes and biopsies per pig (4 OFM probes + 6 biopsies) thus enabling the calculation of the CVs of the repeats.

The question of whether the precision obtained in this study is sufficient to distinguish between two applications in 6 pigs was addressed based on the comparison of two formulation strengths for brepocitinib in Study#1 (brepocitinib cream 3% BID *versus* 0.3% BID) and the comparison of once *versus* twice daily brepocitinib applications in Study#2 (brepocitinib cream 3% QD *versus* 3% BID).

### Reproducibility Between Pig Studies

The reproducibility between studies was evaluated for dOFM only, as biopsies were discontinued following the method assessment in Study#1. The evaluation was based on the comparison of the dOFM data of brepocitinib 3% cream BID and PF-06763809 2.3% solution BID, which were assessed in both studies.

### Verification of dISF Concentration

In Study#2 the dISF concentration of brepocitinib following the brepocitinib 3% cream BID application was assessed at two application sites in two different ways: first via dOFM standard sampling and mathematical correction for the dilution, and second via an recirculation approach directly delivering undiluted dISF (for more details see Hummer *et al*. [45]). For the recirculation approach, the outflow of the dOFM probe was connected to the inflow of the probe via the peristaltic pump tubing such that the perfusate of approx. 30 μL was circulated 16 times in a closed loop for 8 h. Using recirculation, the perfusate becomes fully equilibrated with the surrounding dISF and the drug concentration therein and thus no correction for dilution is required. Three dOFM probes per pig each collected one recirculation sample with a volume of ~ 30 μL after 8 h resulting in three samples of undiluted dISF for direct bioanalysis of the total ISF concentration dISF_tot_.

### Bioanalyses 

Bioanalyses were conducted at JR-HEALTH (Study#1: dOFM samples, biopsy samples, RED samples) and at Unilabs York Bioanalytical Solutions Limited (Study#2: dOFM samples, RED samples). Target analytes were determined by HPLC–MS/MS after protein precipitation. RED was performed at JR-HEALTH. Further details are provided in the Supplementary Material.

## Results and Discussion

### PK Results 

For Study#1 unbound dISF drug concentrations *versus* time profiles are shown in Fig. 3 for two investigational and four prescription drugs (brepocitinib in 2 strengths, tofacitinib, crisaborole, diclofenac, PF-06763809 and PF-06263276). Mean dOFM probe depth for Study#1 was 1.45 ± 0.27 mm, i.e., sampling occurred in the lower dermis.

**Fig. 4:**
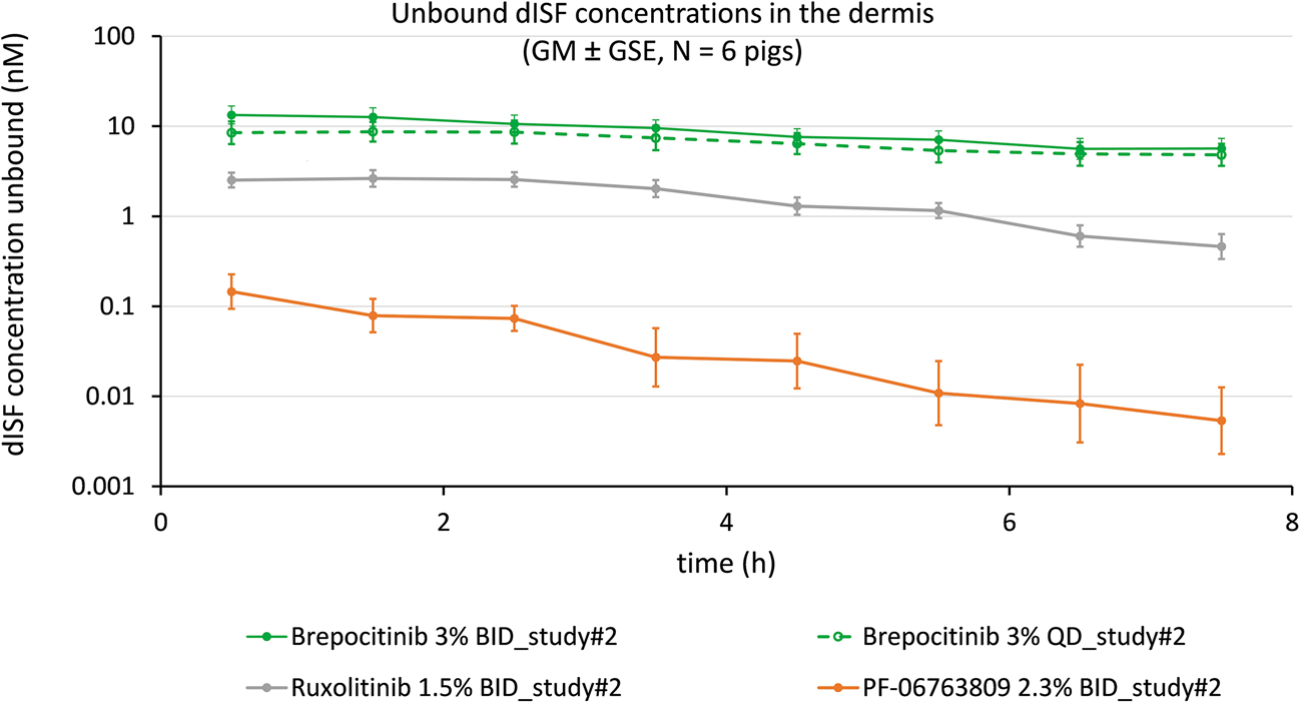
Dermal PK profiles of four drugs in Study #2. Drugs included brepocitinib BID, brepocitinib QD, PF-06763809 and ruxolitinib

For Study#2 unbound dISF drug concentrations *versus *time profiles are shown in Fig. 4 for three investigational drugs (brepocitinib BID, brepocitinib QD, PF-06763809) and one prescription drug (ruxolitinib). The mean dOFM probe depth for Study#2 was 1.57 ± 0.26 mm, i.e., sampling occurred in the lower dermis.

The unbound dISF drug concentrations are also given as averaged concentrations 0–8 h in Table II. This highly condensed view of averaged results enables a direct comparison of all dOFM-derived data (dISF total and unbound concentrations) to the respective biopsy data of all seven drugs in synopsis with the drugs’ molecular properties and clinical efficacies.

**Table II:** Summary of dOFM and Biopsy Data With Selected Background Information

Drug	MW [Da]	cLogP	Fu*	dOFM avg [nM]	dISFtot avg [nM]	dISFu avg [nM]	Biopsy upper dermis [μM]	Biopsy lower dermis [μM]	Main evidence of clinical efficacy
Diclofenac 2%	296	4.3	0.01	31.0	**301**	**3.0**	74.8	2.5	Rx osteoarthritis pain [46]
Crisaborole 2%	251	2.6	0.04	11.2	**99**	**4.2**	29.2	2.5	Rx atopic dermatitis [47]
Brepocitinib 3%	389	1.6	0.68	3.1	**10**	**6.9**	12.8	1.3	Atopic dermatitis Ph2 [48]
Ruxolitinib 1.5%	306	2.5	0.16	1.6	**10**	**1.7**	n.a	n.a	Rx atopic dermatitis and vitiligo [49]
Tofacitinib 2%	312	1.5	0.75	1.4	**4.3**	**3.3**	2.9	0.2	Atopic dermatitis Ph2 [50]
PF-06763809 2.3%	498	3.8	0.05	0.2	**1.8**	**0.1**	3.1	0.2	Failed psoriasis plaque test [51]
PF-06263276 4%	567	~ 4.0	~ 0.001	0.1	**1.1**	**0.001**	2.9	0.2	Failed psoriasis plaque test [52]

Fu* refers to the fraction of unbound drug assessed by rapid equilibrium dialysis (RED) in dOFM samples from pigs. The data for all drugs in Study#1 are shown. Ruxolitinib was added from Study#2 (no biopsies). The drugs from Pfizer and their clinical efficacies were blinded until finalization of data analysis. Concentration data are geometric means (dISFtot/dISFu average 0–8 h: N = 12 profiles for brepocitinib, N = 6 profiles for others; biopsies at 8 h: N = 36 for brepocitinib, N = 18 for others). Bold letters indicate dISF concentrations which are considered most relevant for local drug efficacy. Note: dISFu concentrations are from lower dermis thus representing an underestimate of the concentrations in the upper dermis and epidermis.

### dOFM has Precision to Discriminate Treatments

In Study#1, the evaluation of the precision showed that dOFM results were less variable than biopsy results, i.e., the within-subject dOFM results were more precise than the within-subject results of the biopsy methodology (CV 18–32% *versus* CV 29–54%, p < 0.01, t-test). The Supplementary Material provides the detailed results of the precision and variability evaluation in Table 3 and Table 4 along with an extensive discussion.

A sufficient intra-subject precision is a prerequisite for reliable head-to-head comparisons of treatments (drugs, formulations, doses, etc.) in a study with a limited number of subjects or animals. In Study#1, the precision of dOFM was sufficient to clearly discriminate the 0.3% from the 3% brepocitinib cream BID application (Fig. 5 in Supplementary Material). In Study#2 the precision of dOFM was sufficient to distinguish between the QD and the BID applications with 3% brepocitinib cream (Fig. 6 in Supplementary Material). Here, the difference between applications was visible during the initial hours but not at later time points. For the BID application, the first time point in Fig. 6 corresponded to ~ 12 h after dose application in the evening, and 0–1 h after fresh dose application. For the QD application, the first time point corresponded to ~ 24 h after dose application and 0–1 h after fresh dose application. This result is consistent with the expectation that the largest local concentration difference ought to be observed at the initial time points and that the difference should diminish due to the dose applied at t = 0 to both application sites.

Overall, the precision of preclinical dOFM seen in the dOFM pig model seems to agree with the precision of clinical dOFM in its evaluation for topical bioequivalence, where precision enabled both, the discrimination of products and reproduction of results within narrow acceptance limits in 20 subjects [35, 53]. Precision to enable discrimination of products has also been demonstrated for dermal microdialysis when it had been used in pigs [6] and in volunteers [54] for topical bioequivalence testing. Although microdialysis uses probes with semi-permeable membranes and is more limited in its capacity of sampling large and lipophilic drugs, its principle of continuous sampling of analytes from a linear path in the dermis is comparable to dOFM. As such, the observed precision of the PK measurements seems typical for both methods. What had already been learned in the microdialysis study [6], and is definitely contributing to the dOFM pig model’s high precision and sensitivity for product discrimination, is that the large and homogenous skin areas are available on (domestic) pigs is ideal for placing multiple test sites and sampling probes side-by-side.

### dOFM Pig Model Resulted in Reproducible Data Between Studies

The evaluation of the precision of the methods within each pig showed that dOFM PK data was more precise than biopsy PK data. This should mean that applications can be discriminated in a head-to-head evaluation in the pig model when using dOFM. It is also desirable that data can be reproduced in subsequent pig studies, such that dOFM data can be compared between different pig studies. Therefore, for evaluation of the between-study reproducibility, two drugs were carried forward from Study#1 to Study#2.

The application of brepocitinib 3% cream BID resulted in dISF unbound concentrations of approximately 10 nM in both studies (Fig. 3 and Fig. 4, Fig. 7 in Supplementary Material show the direct comparison). The application with PF-06763809 2.3% solution BID resulted in dISF unbound concentrations of approximately 0.1 nM in both pig studies (Fig. 3 and Fig. 4, Fig. 8 in Supplementary Material show the direct comparison). That is, the results for those two investigational drugs were rather consistent- and so was the clear concentration difference of 2 magnitudes between the two drugs.

The results suggest that the reproducibility of the dOFM pig model between studies is acceptable. 

### dISF Concentration was Verified by Recirculation

The dISF concentration for brepocitinib 3% BID, which was calculated from the dOFM sample considering the RRs for the unbound and the protein-bound drug fractions (see the equations in methods section), was successfully verified by using recirculation in Study#2. Recirculation of the perfusate in the dOFM probes (16 recirculations in 8 h) delivered dOFM sample concentrations at 8 h that were similar to the calculated dISF_tot_ concentrations (GM dISF_tot_ was 6.21 nM by recirculation *versus* 8.32 nM calculated, p = 0.6, Mann–Whitney test, Fig. 9 in Supplementary Material).

#### dISF Results for 7 Drugs

The concentration profiles in Figs. 3 and 4 show decreasing dISF concentrations for all drugs at the level of the dermis. These profiles are in line with the profiles obtained for other drugs in the pig dOFM model after 1 week of daily topical treatment using small clinical topical doses (confidential data). This means, at steady-state, a further small dose on pretreated skin on day 7 does not lead to a detectable immediate increase in the dermis. dISF profiles reflect the steady-state concentrations after repeated dosing and the drug elimination phase. These dOFM data seem to agree with the evidence for steady-state conditions summarized in a textbook already in the 1980s [55], which described the slow drug influx and efflux from the skin reservoirs into the skin under steady-state conditions, and listed the preclinical and clinical validation studies showing constant concentrations in different skin layers using various application frequencies.

The average dOFM concentrations from 0 to 8 h, and the average total and the unbound dISF concentrations dISF_tot_ and dISF_u_ as well as the biopsy concentrations at 8 h are provided in Table II. The investigational and prescription drugs are listed according to their dermal interstitial fluid concentrations from high to low dISF_tot_ concentrations to facilitate a direct comparison with the biopsy concentrations in conjunction with background information on the drugs’ molecular properties and clinical efficacy study outcomes.

A requirement for successful topical treatment is, that the drug needs to effectively penetrate the skin barrier, the stratum corneum. In general, the drug’s molecular properties together with how it is formulated should determine its ability to penetrate the skin [56]. Matching this assumption the two investigational drugs (PF-06763809 and PF-0623276) which possess less favorable molecular properties for topical treatment (high MW, high logP and low fraction unbound) clearly showed the lowest dISF levels (dISF_tot_, dISF_u_). Dermal biopsies also showed low concentrations for these two investigational drugs relative to the other drugs, but the biopsy concentrations were not clearly different from the results for some other drugs. We do not discuss the quality of the relationship of dOFM and biopsy data to the molecular properties and clinical efficacies of drugs used in these studies. This is because of the fact that each drug in this study was formulated with a different vehicle and a different strength, such that formulation remains a confounding factor when assessing the relative penetration of the studied drugs.

#### Biopsy Results for 7 Drugs

The dermal biopsy concentrations for the upper dermis and the lower dermis are provided in Table II. Biopsy data are presented in μM as they were significantly higher than dISF_tot_ and dISF_u_ concentrations. These data are in line with a previous study [39] that reported dermal ruxolitinib concentrations of 63 μM (total) and 12 μM (unbound) after topical application of a 1.5% cream formulation. The authors are not aware of other biopsy data published on these drugs to enable a comparison with the dermal biopsy data obtained in the pig model.

The superficial or upper sections of the biopsies represent a mean depth of ~ 0.45 mm (dermis from 0.1 – 0.8 mm in depth), while the lower section has a mean depth of ~ 1.35 mm (dermis from 0.8–1.9 mm in depth). The average ratio for the upper/lower dermis biopsy concentration was ~ 11, which is consistent with the intradermal concentration gradient of approximately 1 log per mm (i.e., factor ~ 10 per mm) reported for topically applied drugs from theoretical and experimental studies [55, 57].

The lower dermis biopsy concentrations were at least one order of magnitude higher than dISF_tot_ concentrations (Table II). Further, as shown above, biopsy concentrations were more variable compared to dOFM concentrations. Researchers have speculated that conventional skin biopsies were often contaminated with residual topical drug calling that the “skin surface contamination issue” [5] and that biopsy concentrations did not consider skin binding [32], or that biopsies were confounded by the high drug concentrations entrapped within skin appendages such as hair follicles and sebaceous glands [55, 58–60]. Recent studies by novel imaging techniques such as MALDI-MSI showed significant drug concentrations in skin appendages that could be responsible for higher and variable biopsy concentrations [61]. The presence of drugs in skin appendages and local release to the dermis should theoretically also affect dOFM data [53], but it does not affect dOFM data to the same degree that biopsies are affected.

### Different Efficacies of the Studied Drugs in Psoriasis *versus *Atopic Dermatitis

Seven different compounds were selected for topical dose administration to cover a range of drug properties (MW, cLogP, protein binding) and to have evidence available on clinical and pharmacological activity to facilitate a very basic comparative assessment of pig PK data of both methods to the drugs’ clinical activity. Drugs with higher dISF concentrations showed clinical efficacy in atopic dermatitis while the two drugs with the lowest dISF concentrations did not show clinical efficacy in the psoriasis plaque test (Table II).

The different efficacies observed for some drugs in psoriasis and atopic dermatitis may also be explained in part by the different pathophysiological skin conditions which should result in different local drug exposures.

The topical drug delivery rate to psoriatic skin is threefold lower compared to atopic dermatitis skin, due to the reduced barrier function of the stratum corneum in atopic dermatitis compared to the intact barrier function in psoriasis plaques [4]. Moreover, drug clearance rates are threefold higher in psoriatic lesions, due to the higher capillary blood perfusion rates of psoriatic skin lesions compared to AD lesions. Taken together, Trottet [4] concluded, that the treatment of psoriatic skin requires a topical drug product that demonstrates a ~ tenfold increased drug flux rate (to compensate for barrier and clearance) to achieve similar concentration of the drug used to treat atopic dermatitis skin [4].

These additional pathophysiological considerations of psoriatic lesions may help explain why PF-06263276 and PF-06763809 have failed in psoriasis studies. Both drugs showed relatively low dISF concentrations probably due to their less favorable molecular properties (high MW, high cLogP, low unbound fraction f_u_) despite an infinite dose application in the clinical study to maximize penetration of the hyperkeratinized psoriatic skin barrier [51, 52]. Also consistent with Trottet’s observations, oral brepocitinib [62] and oral tofacitinib [63] showed clear clinical efficacy in psoriasis but the efficacy of their respective topical formulations in psoriasis was modest [50, 64]. In addition, the possibility remains that the compound action in the systemic compartment could be an important component to achieve efficacy in psoriasis for these JAK inhibitors.

A more detailed summary of clinical efficacy results of these drugs with cited references is included in the Supplementary Material.

### Benefits and Limitations of the dOFM Pig Model

Methods such as skin flux (*in vitro* permeation tests, IVPT) and skin sampling (biopsies and dOFM) have been used to predict skin permeation of topical drugs. However, IVPT lacks dermal clearance and the ability to assess the free drug concentration which limits IVPT’s direct use in correlating drug concentration with target modulation and clinical efficacy. Biopsies, as seen in this and other studies, allow a gross evaluation of the total amount of drug that has penetrated but are limited by an increased contamination (risk) that results in high variability and inability to distinguish free from bound drug concentrations. In contrast, dOFM allows assessment of free and total drug concentrations close to the dermal target site in an *in vivo* model with dermal clearance.

The most relevant limitation of the current dOFM pig model is that dOFM assesses the drug at the level of the mid to low dermis. As such, projecting the unbound dISF concentration at more superficial target sites (e.g., lower epidermis and upper dermis) based on dOFM sampling at mid to lower dermis would likely underestimate the concentration by a factor of ~ 10 (for upper dermis) to ~ 100 (for epidermis). A correction by factor 10 to extrapolate the concentration from the probes in the lower dermis (~ 1.5 mm) to the upper dermis (~ 0.4 mm) seems reasonable considering the concentration gradients found within the dermis [55, 57] and the ratios between upper/lower biopsies in pig Study#1. But this factor may be over-simplistic and may not be valid for all drugs and skin conditions. Further effort is needed to understand dermal concentration gradients in normal *versus* disease skin for bound and unbound drugs.

### How to Further Improve and Utilize the dOFM Pig Model?

A future direction will be the establishment of a model that is able to reliably project dOFM concentrations in the lower dermis to dISF_u_ concentrations at target site(s) of action in upper dermis and lower epidermis. Together with knowledge of relevant pharmacological activity (e.g., IC50) this enables assessment of the likelihood of clinical success for the applied drug/formulation.

Beyond the use of dOFM for verifying dermal tissue exposure in the preclinical stage, dOFM can also be carried forward to early clinical proof-of-concept microplaque studies to verify the intended local PK-PD directly in patients for topical drugs [37, 38, 65] or antibodies [36, 66–68]. Thus, preclinical and clinical dOFM studies may support “Quick-Win-Fast-Fail” approaches and the new paradigms of testing and decision making to improve research and development productivity.

## Conclusion

The evaluation of dOFM in an *in vivo* pig model indicated acceptable intra-study precision to distinguish between different drugs and different dosing frequencies; and the model also indicated reproducibility between studies. We conclude that the dOFM pig model provides a reliable assessment of drug concentrations in dISF following topical application. Biopsy concentrations were generally higher and more variable than dOFM derived interstitial concentrations due to possible contributions from drugs bound to tissue and drugs associated with skin appendages.

### Supplementary Information

Below is the link to the electronic supplementary material.

Supplementary file1 (PDF 1.15 MB)
