# Correction: Comparative Study of Dermal Pharmacokinetics Between Topical Drugs Using Open Flow Microperfusion in a Pig Model

**DOI:** 10.1007/s11095-024-03659-5

**Published:** 2024-01-19

**Authors:** Manfred Bodenlenz, Thean Yeoh, Gabriel Berstein, Shibin Mathew, Jaymin Shah, Christopher Banfield, Brett Hollingshead, Stefanus J. Steyn, Sarah M. Osgood, Kevin Beaumont, Sonja Kainz, Christian Holeček, Gert Trausinger, Reingard Raml, Thomas Birngruber

**Affiliations:** 1 HEALTH - Institute for Biomedical Research and Technologies, Joanneum Research Forschungsgesellschaft M.B.H, Neue Stiftingtalstrasse 2, 8010 Graz, Austria; 2 Pfizer Research Technology Center, 1 Portland St, Cambridge, MA 02139 USA

**Correction: Pharmaceutical Research**

10.1007/s11095-023-03645-3

The original article has been corrected to update the author list back to the submitted author list. This error was introduced during production, and not the fault of authors.

