# Supplementary material for: Comparative Study of Dermal Pharmacokinetics Between Topical Drugs Using Open Flow Microperfusion in a Pig Model
Source: Pharm Res. 2023 Dec 29;41(2):223–34. doi: 10.1007/s11095-023-03645-3 (PMC10879402; doi:10.1007/s11095-023-03645-3)
Supplement: Supplementary file 1 — Supplementary file1 (PDF 1.15 MB) [file 11095_2023_3645_MOESM1_ESM.pdf]

Supplementary Material

Comparative study of dermal pharmacokinetics between topical drugs using open flow microperfusion in a pig model

Manfred Bodenlenz[1], Thean Yeoh[2], Gabriel Berstein[2], Shibin Mathew[2], Jaymin Shah[2], Christopher Banfield[2], Brett Hollingshead[2], Stefanus J. Steyn[2], Sarah M. Osgood[2], Kevin Beaumont[2], Sonja Kainz[1], Christian Holeček[1], Gert Trausinger[1], Reingard Raml[1], Thomas Birngruber[1]

[1] HEALTH – Institute for Biomedical Research and Technologies, Joanneum Research Forschungsgesellschaft m.b.H, Neue Stiftingtalstrasse 2, 8010 Graz, Austria

[2] Pfizer Research Technology Center, 1 Portland St, Cambridge, MA 02139, USA

Summary of clinical efficacy of selected drugs

Ruxolitinib, brepocitinib, tofacitinib, and PF-06263276 are inhibitors of JAK family of enzymes, which transduce signals from cytokine receptors [69].

Ruxolitinib is a JAK1/2/3 inhibitor which is approved as a topical therapy for atopic dermatitis and vitiligo at 1.5% in a cream formulation (Incyte, Opzelura, prescribing information, 2022). In addition, efficacy in other dermatological diseases such as psoriasis has been reported (reviewed in [70]).

Brepocitinib is a JAK1/TYK2 inhibitor. Topical brepocitinib (cream formulation) was reported to be efficacious in an atopic dermatitis clinical study [48] where 1% formulation met the primary endpoint and 0.1%, 0.3%, and 3% formulations met the key secondary endpoint. In a psoriasis clinical study, topical brepocitinib 0.1%, 0.3%, 1% and 3% did not meet the primary and secondary endpoints but showed numerical beneficial trends [64].

Tofacitinib is a JAK1/3 inhibitor. Topical tofacitinib (2% ointment formulation) was efficacious in an atopic dermatitis clinical study [71]. This formulation was also reported to be efficacious in psoriasis clinical studies at week 4 [72] and week 8 but not at week 12 [50]. Tofacitnib has been investigated by Handler et al. 2021 [33] in the dOFM pig model finding approximately 10-fold higher concentrations. However, that data are not comparable to our study due to significant differences in study protocols, dosing, and formulations which were different from the clinical formulation.

PF-06263276 is a JAK1/2/3 inhibitor. PF-06263276 in the 4% solution formulation described in this study showed no efficacy in a human psoriasis plaque test [52].

PF-06763809 is a retinoic acid-related orphan receptor variant 2 (aka, RORγt) inhibitor which is a transcription factor required for T helper 17 cell differentiation [73]. PF-06763809 at 2.3% in the formulation used in this study showed no efficacy in a human psoriasis plaque test [51].

Crisaborole is a phosphodiesterase-4 inhibitor. Topical crisaborole (2% ointment) is approved as a topical therapy for atopic dermatitis (Pfizer, Eucrisa, prescribing information 2020). In addition, there are reports of its efficacy in other dermatology diseases [74].

Diclofenac is a cyclooxygenase-1 and -2 inhibitor that is indicated as topical therapy for osteoarthritis pain (Novartis, Voltaren, prescribing information, 2009 [46]).

New references:

69. Philips RL, Wang Y, Cheon HJ, Kanno Y, Gadina M, Sartorelli V, et al. The JAK-STAT pathway at 30: Much learned, much more to do. Cell. 2022;185(21):3857–76. https://doi.org/10.1016/j.cell.2022.09.023

70. Tegtmeyer K, Ravi M, Zhao J, Maloney NJ, Lio PA. Off-label Studies on the Use of Ruxolitinib in Dermatology. Dermatitis. 2021;32(3):164–72. https://doi.org/10.1097/der.0000000000000658

71. Bissonnette R, Papp KA, Poulin Y, Gooderham M, Raman M, Mallbris L, et al. Topical tofacitinib for atopic dermatitis: a phase IIa randomized trial. Br J Dermatol. 2016;175(5):902–11. https://doi.org/10.1111/bjd.14871

72. Ports WC, Khan S, Lan S, Lamba M, Bolduc C, Bissonnette R, et al. A randomized phase 2a efficacy and safety trial of the topical Janus kinase inhibitor tofacitinib in the treatment of chronic plaque psoriasis. Br J Dermatol. 2013;169(1):137–45. https://doi.org/10.1111/bjd.12266

73. Huang W, Littman DR. Regulation of RORγt in Inflammatory Lymphoid Cell Differentiation. Cold Spring Harb Symp Quant Biol. 2015;80:257–63. https://doi.org/10.1101/sqb.2015.80.027615

74. Makins C, Sanghera R, Grewal PS. Off-Label Therapeutic Potential of Crisaborole. J Cutan Med Surg. 2020;24(3):292–6. https://doi.org/10.1177/1203475420909794

Skin Biopsies procedure

After the termination of 8 h dOFM sampling, the epidermis was completely removed by heat separation and three 6 mm punch biopsies were taken from each application site. To avoid contamination a new disposable biopsy punch, scalpel blade and forceps were used for each single dermis biopsy. Prior to analysis, the frozen dermal samples were split into upper and lower dermis.

The biopsy procedure in brief:

- <u>Cleaning of skin to remove the remaining test products</u>: The application sites were cleaned by using a gauze pad soaked with water. Afterwards the application sites were dried with a gauze pad and shaved once more using disposable razors.
- <u>Heat separation to remove the epidermis from the application sites</u>: A heated steel block (60°C) was gently pressed on the skin for 60 seconds. Afterwards the epidermis was cut at the margin of the heated site using a scalpel and the epidermis was removed using forceps.
- <u>Taking skin biopsies</u>: Three skin biopsies were taken using 6 mm biopsy punches and disposable forceps. The adipose tissue was removed using a scalpel and the skin sample was put into an empty, weighed, labelled cryotube. The filled tube was weighed again and snap frozen in liquid nitrogen.
- <u>Prior to bioanalysis each skin biopsy was divided into 2 layers at JR-HEALTH</u>: Using cooled forceps and a scalpel the frozen samples were cut into a superficial skin section and a deep section, by cutting at a depth of ~0.8 mm (superficial section approx. 0.1-0.8 mm, deep section approx. 0.8-2 mm). The two newly formed samples were put into two prepared cryotubes, labeled for superficial biopsies (s) and deep biopsies (d). 54 sections per pig were homogenized and drug concentration was determined according to the SOP in the laboratory.

Rapid Equilibrium Dialysis -RED

Standard Protocol: A RED device with a single-use plate for 48 samples with 8K MWCO inserts (Thermo Scientific Pierce, Rockford, IL, USA) was used. 50 µL of the drug-spiked protein sample were added to each plasma chamber and 300 µL of PBS buffer were added to each buffer chamber. Each plate was sealed with a polyester sealing mat (VWR International, Radnor, PA, USA) and incubated at 37 ± 1°C (Binder GmbH, Tuttlingen, Germany) while agitating at 300 rpm on an orbital shaker (Heidolph Instruments, Schwabach, Germany) for 8 h. After 8 h, a sample of approximately 50 µL was collected from each chamber for analysis. For each sample the free and bound fractions were calculated according to following formulas:

% free drug fraction = c(buffer chamber)/c(plasma chamber) *100

% bound drug fraction = 100 - % free drug fraction

For each tested compound, the time to equilibrium (2,4,6,8 h) as well as binding behaviour at 3 different concentration (500, 1000, 2000 nM) and in different matrices (pig serum, diluted pig serum (pig serum was diluted 1:1 with Elomel), and OFM-perfusate) was tested. The concentration dependence was not considered as critical for the determined concentration range and the investigated matrices. For some drugs such as ruxolitinib, species differences of protein binding were noted between pig matrix and the perfusate (containing human albumin). The protein binding used for RR calculation was assessed for each compound at 25 nM in OFM matrix, which was obtained from an existing stock of dOFM matrix.

Bioanalyses Details

Bioanalyses were done at JR-HEALTH (study#1, dOFM, biopsy and RED samples) and at Unilabs York Bioanalytical Solutions Limited (study#2, dOFM samples and RED samples).

Study#1

For analysis to 30 µL OFM samples, calibration standards (0.3 - 250 ng/mL), or quality controls (0.3 ng/mL, 70 ng/mL, 200 ng/mL) or 20 µL of the RED samples, 70 µL 0.1% formic acid in acetonitrile (v/v) including 40 ng/mL internal standard (ISTD) were mixed in 0.5 mL low bind Eppendorf tubes and incubated at 4°C for 20 min. Tubes were centrifuged at 13,000 g for 10 min. Then supernatants were transferred to a 0.2 mL 96-well plate, 40 µL of MilliQ water were added and injected into the HPLC-MS.

Skin biopsies were homogenized, in 2 mL reinforced tubes containing four metal beads (2.38 mm) and 500 µL 50% acetonitrile/water 0.1% formic acid (v/v) with 3 cycles of 30 seconds at 8.0 m/s. In between the runs, the samples were cooled on wet ice for approx. 2 min. After homogenization 1 mL of acetonitrile was added to the homogenate and the mixture was centrifuged at 17,000 g for 10 min. To 30 µL of the supernatant, 70 µL 0.1% formic acid in acetonitrile (v/v) including 40 ng/mL ISTD standard and 40 µL of water were added, mixed and injected into the HPLC-MS.

The samples were analyzed with an UHPLC 1290 Infinity II system coupled with a 6495B QqQ mass spectrometer, Agilent Technologies, Santa Clara, CA, USA). Chromatographic separation was achieved using an Acquity UPLC column HSS T3, 2.1 mm x 75 mm, 1.8 µm (Waters Corporation, Milford, CT, USA) and a gradient elution with two mobile phases [mobile phase A: ammonium acetate 1.3 M: oxalic acid 10 mg/mL : water : acetic acid (0.1 : 0.16 : 100 : 0.1 v/v), mobile phase B acetonitrile 0.1% (v/v) formic acid. The applied flow rate was 500 µl/min, the injection volume 10 µl, and the column temperature was 35°C. The multistep gradient started with 10% mobile phase B for 0.5 min, increased to 90% mobile phase B within 3.5 min, and was finally maintained at 90% for 0.5 min prior to changing again to 10% mobile phase B. For the analysis of diclofenac the same chromatographic method was used with following mobile phases: [mobile phase A: 0.1% formic acid in Milli-Q water (v/v); mobile phase B: 0.1% formic acid in

acetonitrile (v/v)]. Mass detection was performed in positive and negative ionization (PFJR005 and diclofenac) MRM mode (PFJR-001[brepocitinib]: 390.2 → 340, PFJR-001_ISTD: 395.2 → 345.4, PFJR-002 [PF-06763809]: 498.2 → 317.3, PFJR-002_ISTD: 504.4 → 323.4, PFJR-003 [PF-06263276]: 567.0 → 190.0, PFJR-003_ISTD 577.0 → 200.0, PFJR-004 [tofacitinib]: 313.2 → 173.0, PFJR-004_ISTD 317.2 → 173.2, PFJR-005 [crisaborole]: 322.0 → 250.0, PFJR-005_ISTD: 326.0 → 254.0, diclofenac: 294.0 → 250.0, diclofenac_ISTD: 300.0 → 256.0).

The lower limits of quantification (LLOQs) obtained for OFM and biopsy samples were in the range 0.25 – 0.5 ng/mL or approximately 0.5 - 1 nM, respectively (study#1). The LLOQs for the target analytes refer to the nominal concentrations of the lowest calibration standard within the acceptance criteria.

Study#2

Calibration standards (0.25 – 2500 nM for PF-06763809 and brepocitinib; 0.250 – 1500 nM for ruxolitinib) and quality controls (QCs) (1, 5, 200 and 1500 nM) were prepared in OFM perfusate. For analysis, 10 µL of OFM sample, calibration standard or QC was combined with 10 µL ISTD in acetonitrile:water (25:75, v/v). Then 200 µL of acetonitrile was added after which the samples were vortexed and centrifuged at 3000 rpm for 10 min. The supernatants (100 µL for PF-06763809 and brepocitinib; 50 µL for ruxolitinib) were transferred to a 96-well plate and 100 or 250 µL of acetonitrile:water (50:50, v/v) was added, respectively. The ISTD used for PF-06763809 and brepocitinib analysis was fluconazole (75 ng/mL). The ISTD used for ruxolitinib was propranolol (60 ng/mL).

The samples were analyzed using a 2-Dimensional LC system coupled with an AB Sciex 6500+ (PF-06763809 and brepocitinib) or 5000 (ruxolitinib) mass spectrometer. The loading column used was a Halo Fused-core 2.7µ C18 100Å (50 x 2.1 mm ID) with mobile phase A consisting of 90% water, 10% methanol (containing 0.1% trifluoroacetic acid). Chromatographic separation was achieved using the eluting column Kinetex SB 2.6µ C18 100Å (50 x 2.1 mm ID) and a gradient elution with two mobile phases (mobile phase A: 5 mM ammonium acetate (containing 0.075% formic acid) and mobile phase B: acetonitrile (containing 0.1% formic acid). Chromatography was performed at a flow rate of 1.0 mL/min (PF-06763809 and brepocitinib) or 0.7 mL/min (ruxolitinib) with a column temperature of 60°C and an injection volume of 10 µL. For PF-06763809 and brepocitinib, the gradient started with 10% mobile phase B for 0.4 min, ramped to 90% B over 1 min, maintained 90% B for 1 min, ramped to 10% B for 0.05 min, then held at 10% for 0.55 min. For ruxolitinib, the gradient started with 10% mobile phase B for 0.45 min, ramped to 90% B over 1.55 min, maintained 90% B for 0.7 min, ramped to 10% B over 0.1 min, then held at 10% B for 0.7 min. Mass spectrometry detection was performed in positive ionization MRM mode. The following transitions were monitored, PF-06763809: 498.4→317.2; brepocitinib: 390.3→340.2; ruxolitinib: 307.1→186.1; fluconazole: 307.2→238.1; and propranolol: 260.3→116.2.

RED Samples for ruxolitinib

OFM perfusate samples generated from the RED study for ruxolitinib were assayed as described above for study#2. The calibration standard range used was 5 – 2500 nM with QCs at 5, 200 and 1500 nM. The analysis of the RED samples for the other compounds has been described in the Bioanalytics section on study#1.

Results Details - Which method is more precise?

This evaluation investigated for each method the deviations between the mean results of the two test sites, which were treated with the same product, and the evaluation considered the two sites as "repeats". This was required, as the dOFM sample at 8 h in each site was a pooled sample (i.e. already a mean) of the two adjacent probes, thus saving analytical costs. To treat biopsies similar in the evaluation, we calculated the mean of the adjacent biopsies and subjected the means to the evaluation.

Table 3 and Table 4 provide the coefficients of variation (CV) for dOFM and for biopsies within each subject (pig) in study#1, separately for the 0.3% brepocitinib cream (2 test sites) and the 3% brepocitinib cream (2 test sites).

dOFM results were less variable than biopsy results, i.e., the within-subject dOFM results were more precise than the within-subject results of the 'improved' biopsy methodology ($p<0.01$, t-test). This observation was unexpected, as the biopsy procedure included the complete epidermis removal prior to the collection of the dermis in order to reduce contamination of the dermis samples by high epidermal concentrations. Moreover, to compare the methods at the same time point (8 h), the 8 h dOFM pooled sample result (1 analysis) was subjected to CV% analysis, which should be associated with more bioanalytical uncertainty than a mean of biopsy results.

The increased variability/reduced precision observed with biopsies is in line with earlier reports [32]. Biopsies may be more affected than dOFM results by the high drug concentrations residing in hair follicles [32,75] and sebaceous glands [61] following a period of BID treatment. Interestingly, the CVs for the lower dermis biopsies results tended to be higher than those for the upper dermis (n.s., t-test). This finding suggests that deeper structures such as hair follicles may be contributing to the variability.

Table 3 Intra-Subject variability of log-transformed dOFM results expressed as CV%

	P1	P2	P3	P4	P5	P6	CV% mean	CV% median
%CV dOFM for 0.3% cream	25%	18%	12%	18%	18%	20%	**18%**	18%
%CV dOFM for 3% cream	45%	28%	26%	22%	59%	11%	**32%**	27%

CVs of log transformed data were calculated using the equation of Nelson et al. [43,44]. The means for non-transformed data are 18% and 30% for 0.3% and 3% cream, respectively.

Table 4 Intra-Subject variability of log-transformed biopsies expressed as CV%

	P1	P2	P3	P4	P5	P6	CV% mean	CV% median
for 0.3% cream								
%CV lower dermis biopsy	39%	62%	96%	53%	35%	38%	**54%**	46%
%CV upper dermis biopsy	100%	51%	19%	24%	35%	22%	**42%**	29%
for 3% cream								
%CV lower dermis biopsy	24%	77%	84%	33%	26%	56%	**50%**	44%
%CV upper dermis biopsy	27%	56%	34%	21%	27%	10%	**29%**	27%

CVs of log transformed data were calculated using the equation of Nelson et al. [43,44]. The means for non-transformed data are 48/36% and 50/28% for 0.3% and 3% cream, respectively.

Noteworthy, differences in the precision (intra-subject variability) of the methods should also be reflected by differences in the overall CV% being a combined measure of intra- and inter-subject variability (Table 5). However, the dimension of the overall CV should be dominated by the inter-subject variability, which is known to be considerable for any study investigating topical PK, at least in human

topical studies. Still, this evaluation shows a lower CV% for dOFM compared to dermal biopsies.

Table 5 Overall Inter-and Intra-Subject variability of log-transformed data expressed as CV%

	CV% biopsy	CV% dOFM
for 0.3% cream		
lower dermis	124%	34%
upper dermis	122%	-
for 3% cream		
lower dermis	120%	70%
upper dermis	109%	-

CVs of log transformed data were calculated using the equation of Nelson et al. [43,44]. The CVs for non-transformed biopsy data are 92/108% (lower/upper) for 0.3% cream and 88/107% for 3% cream. The corresponding CVs for non-transformed dOFM data are 34% and 87% for 0.3% and 3% cream.

New references:

75. Liu X, Grice JE, Lademann J, Otberg N, Trauer S, Patzelt A, et al. Hair follicles contribute significantly to penetration through human skin only at times soon after application as a solvent deposited solid in man. Br J Clin Pharmacol. 2011 Nov;72(5):768–74. https://doi.org/10.1111/j.1365-2125.2011.04022.x

Results Details - Discrimination of brepocitinib treatments by dOFM

A sufficiently low intra-subject precision is a prerequisite for reliable head-to-head comparisons of treatments (drugs, formulations, doses, etc.) in a study with limited number of subjects or animals. In study#1, the precision of dOFM was sufficient to clearly discriminate the 0.3% from the 3% brepocitinib cream BID treatment (Fig. 5). In study#2 the precision of dOFM was sufficient to distinguish between the QD vs the BID treatments with 3% brepocitinib cream (Fig. 6). Here, the difference between treatments was visible during the initial hours but not at later time points. For the BID treatment, the first time point in Fig. 6 corresponded to ~12 hours after the dose application in the evening, and 0-1 hours after fresh dose application. For the QD treatment on the other hand, the first time point corresponded to ~24 hours after dose application and 0-1 hours after fresh dose application. This result is consistent with the expectation that the largest local concentration difference ought to be observed at the initial time points and that the difference should diminish due to the dose administered at t=0 to both treatment sites.

Overall, the precision of preclinical dOFM seen in the dOFM pig model seems to agree with the precision of clinical dOFM in its evaluation for topical bioequivalence [35,53], where it enabled both the discrimination of products and reproduction of results within narrow acceptance limits in 20 subjects [published for acyclovir products, papers for lidocaine/prilocaine products and for diclofenac products are in preparation]. Precision to enable discrimination of products had also been demonstrated for dermal microdialysis when it was used in pigs [6] and in volunteers [54] for topical bioequivalence. Although microdialysis uses probes with semi-permeable membranes and is more limited in its capacity of sampling large and lipophilic drugs, its principle of continuous sampling of analytes from a linear path in the dermis is comparable to dOFM. As such, the observed precision of the PK measurements seems typical for both methods. What had already been learned in the microdialysis study [6], and definitely contributing to the

pig model's high precision and sensitivity for product discrimination, is the large and homogenous skin area available on (domestic) pigs for placing multiple test areas and sampling probes site-by-site.

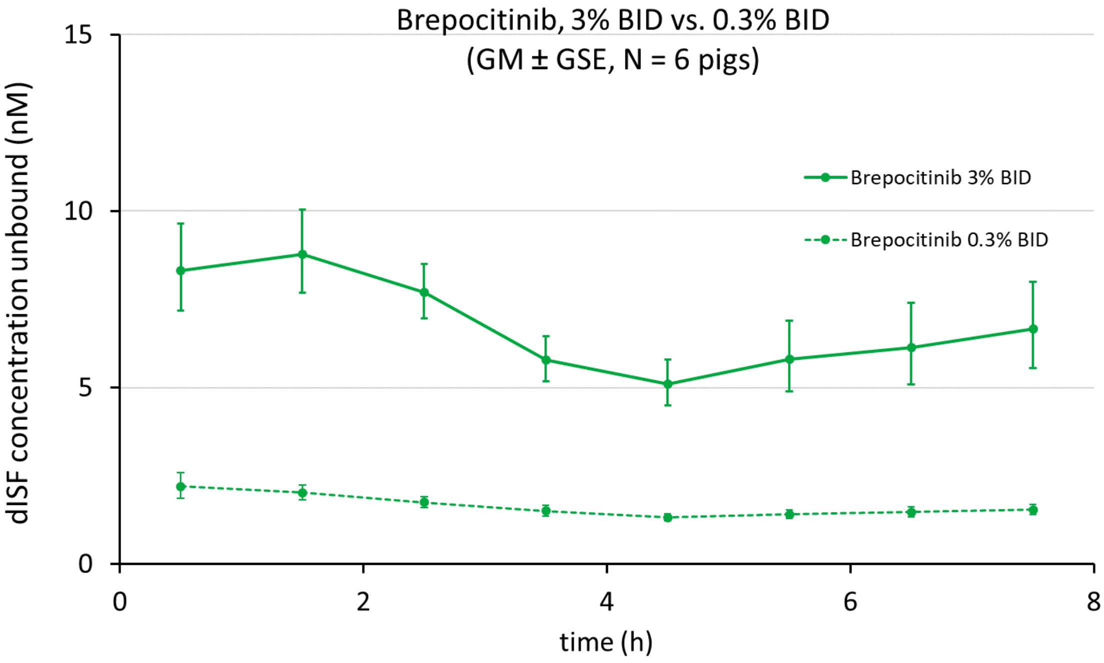

Fig. 5 Brepocitinib 3% BID vs 0.3% BID in study#1

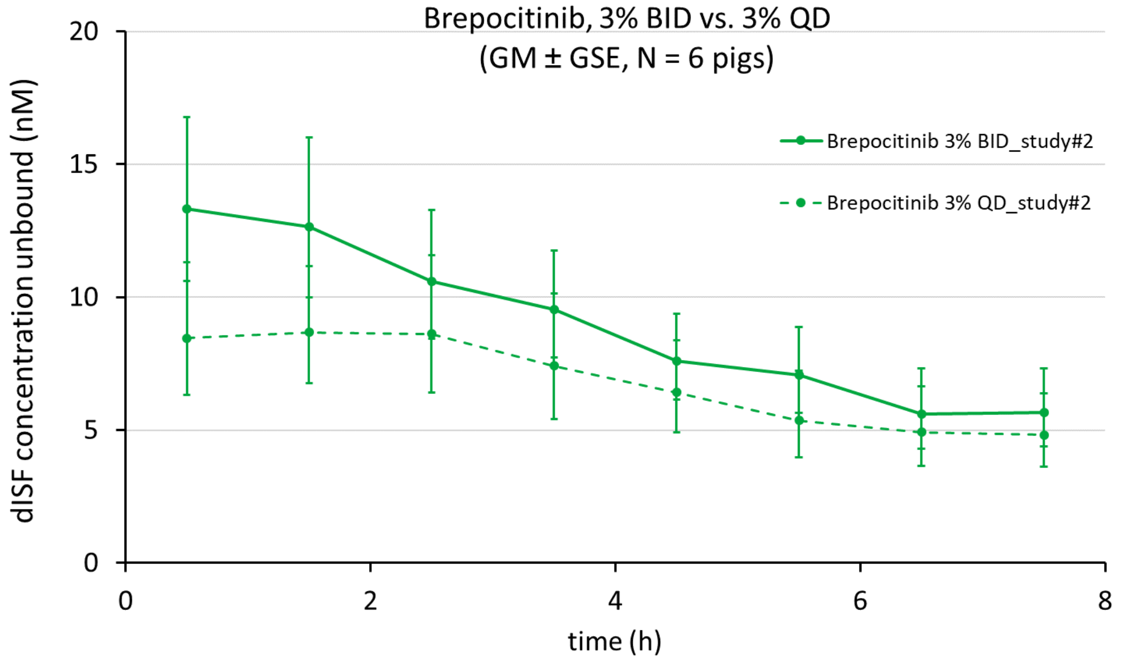

Fig. 6 Brepocitinib 3% BID vs 3% QD in study#2

Results Details - Can the dOFM pig model reproduce data?

The evaluation of the precision of the methods within each pig showed that dOFM PK data were more precise than biopsy PK data. This implies that treatments can be discriminated in the head-to-head evaluation in the pig model when using dOFM. It is also desirable that data can be reproduced in subsequent pig studies, such that dOFM data can be compared between different pig studies. Therefore, for evaluation of the between-study reproducibility, two drugs/treatments were carried forward from pig study#1 to pig study#2.

The treatment with brepocitinib 3% cream BID resulted in dISF unbound concentrations of approximately 10 nM in both studies (Fig. 7). The treatment with PF-06763809 2.3% solution BID resulted in dISF unbound concentrations of approx. 0.1 nM in both pig studies (Fig. 8). The results for those two drugs were rather consistent, with a clear concentration difference of 2 magnitudes between the two drugs.

The deviations from perfect reproduction of dOFM data (means, Geometric means) should be due to the (i) the known-inter subject variability, (ii) the rather small study sizes (6 pigs in each study), and (iii) the fact that only a part of dOFM probes were dedicated to these drugs. Moreover, the samples of the two studies were analyzed in different laboratories using slightly different limit of quantifications, which should explain some of differences between the curves for PF-06763809 2.3% showing very low dISF concentrations.

The data suggest that both the reproducibility between studies and the sensitivity and precision to discriminate treatments within a pig study should be sufficient when using the dOFM pig model, in particular when the study has a clear focus on one or two primary comparisons such that all dOFM probes in a pig can be dedicated to those primary readouts.

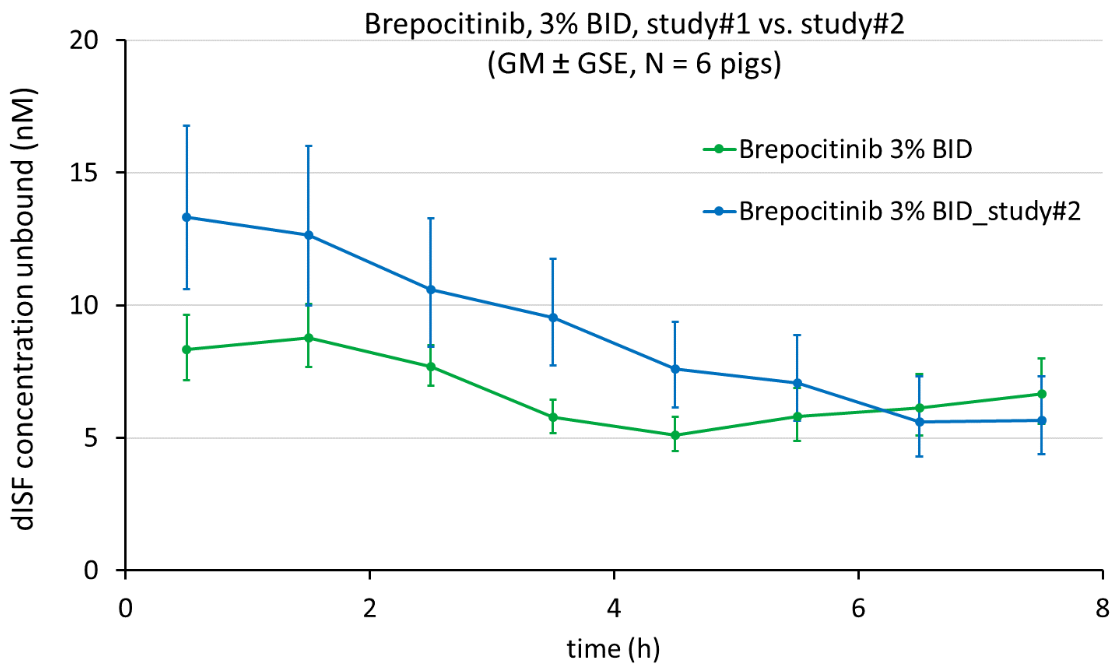

Fig. 7 Dermal ISF unbound of Brepocitinib 3% cream BID study#1 vs study#2, to show reproducibility between studies

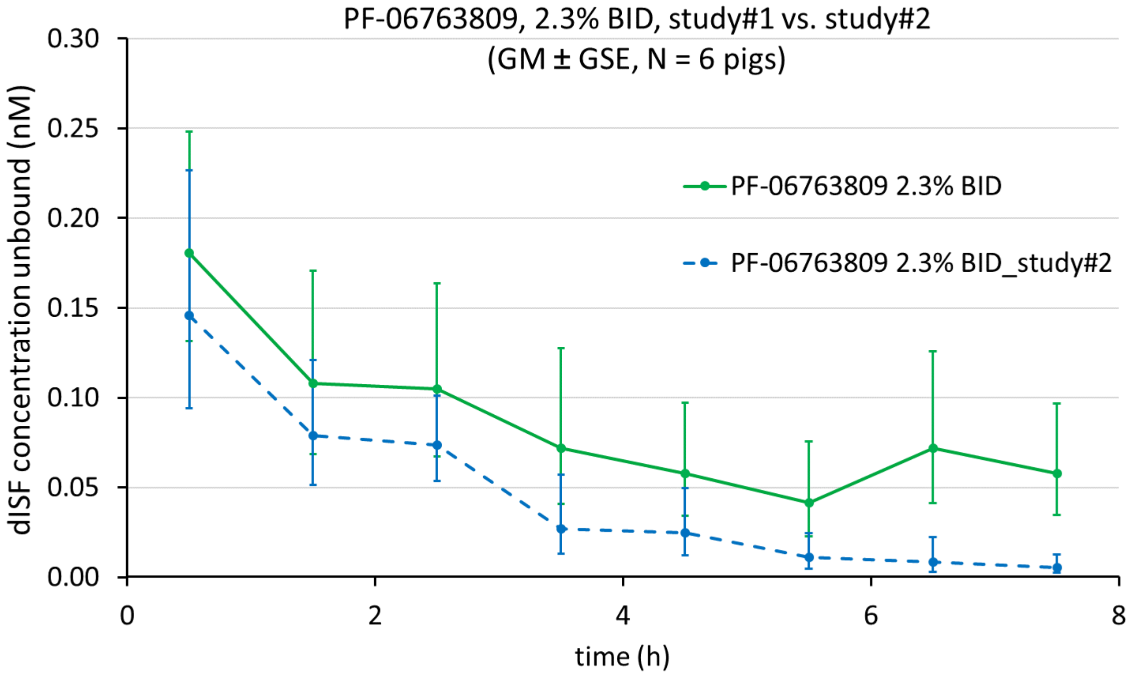

Fig. 8 Dermal ISF unbound of PF-06763809 2.3% solution BID study#1 vs study#2, to show reproducibility between studies

Results Details - Validation of dISF drug concentration

The dISF concentration for brepocitinib 3% BID, which was calculated from the dOFM sample considering the RRs for the unbound and the protein-bound drug fractions (see the equations in methods sections), was successfully verified by the method of recirculation in study#2. Recirculation of the perfusate in the dOFM probes in the extra treatment site (16 recirculations within 8 hours) delivered dOFM sample concentrations at 8 hours that were rather variable but similar to the calculated $dISF_{tot}$ concentrations (Geometric Mean $dISF_{tot}$ was 6.21 nM by recirculation versus 8.32 nM calculated) (Fig. 9).

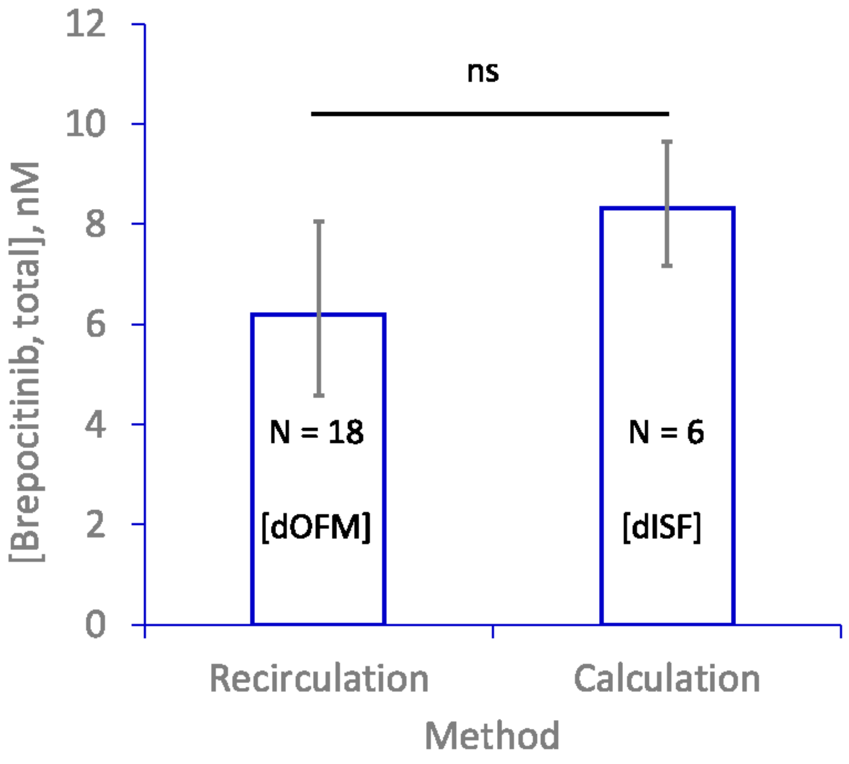

Fig. 9 Comparison of concentration of Brepocitinib (3%) in the probe sample after recirculation vs. projected dISF value from single pass at the end of the study duration (7.5 h). The data indicates GM and GSE. dISF concentration calculated using a RR of 40:10 is similar to the recirculation value (p = 0.6, Mann-Whitney test).
